# Ergonomic learning curves on gynecological laparoendoscopic single-site (LESS) surgery

**DOI:** 10.1186/s12893-023-02241-x

**Published:** 2023-10-27

**Authors:** Ye Yang, Yu Qin Pan, Qi Lu, Wei Bao, Min Wang, Wei Liu, Su Fang Wu

**Affiliations:** 1 Obstetrics and Gynecology Department, Shanghai General Hospital, Shanghai Jiao Tong University School of Medicine, 85 Wujin Road, Hongkou, Shanghai, 200080 P.R. China; 2 Surgery Department, Shanghai General Hospital, Shanghai Jiao Tong University School of Medicine, Hongkou, Shanghai, P.R. China; 3 General Surgery Department, Shanghai General Hospital, Shanghai Jiao Tong University School of Medicine, Hongkou, Shanghai, P.R. China; 4 Educational Department, Shanghai General Hospital, Shanghai Jiao Tong University School of Medicine, 85 Wujin Road, Hongkou, Shanghai, 200080 P.R. China

**Keywords:** Ergonomic posture, Hands-on performance, Gynecologic, Learning and teaching curve, Laparoendoscopic single-site Surgery (LESS)

## Abstract

**Background:**

Few previous studies have introduced general techniques to overcome the “chopstick effect” in laparoendoscopic single-site surgery (LESS). We aim to investigate and highlight the key ergonomic methodologies for gynaecologic LESS based on the surgeon’s hands-on performance.

**Methods:**

The first author surgeon A reviewed and analyzed the LESS procedures performed by herself and how she taught surgeon B from January 2021 to April 2022. The procedures were classified based on technical difficulty and learning periods, and the hands-on technical skills of LESS module were evaluated.

**Results:**

Surgeon A conducted 580 LESS procedures, which were divided into the novice (n = 48) and intermediate (n = 33) periods, and the remaining cases were included in the routine period. We formed a special ergonomic LESS operating methodology: Maintain good LESS laparoscopic spatial sensation, keep hand-eye coordination, well cooperation between the main surgeon and the assistant; Experienced multiport laparoscopy surgery (MPS) skills, improve basic LESS technique: grasp, lift, transfer, place, blunt separating, coagulation and cutting. Coordination location, orientation, movements, and flexion or extension of shoulders, arms, elbow, wrist and finger joints; Maintain strength, tension and ambidexterity postures with joint and muscular efforts to control instruments. Surgeon B learned the above experiences by performing 39 LESS procedures under the guidance of surgeon A.

**Conclusion:**

This educational research sheds light on the common challenges faced in LESS and presents the importance of ergonomic hands-on performance skills in improving surgical outcomes, which could serve as a guide for future training and education in LESS.

**Supplementary Information:**

The online version contains supplementary material available at 10.1186/s12893-023-02241-x.

## Introduction

Laparoscospic single-site surgery (LESS) [1] has been considered as a minimally invasive technique through a single multiport device accessed via a small incision in the skin, characterized by a reduction in the number and size of incisions. The single-site approach has been found to be both feasible and safe, making it suitable for a wide range of gynecologic conventional laparoscopic surgery (CLS) that previously required multiple incisions [2, 3]. Transumbilical laparoendoscopic single-site (Tu-LESS) surgery offers several benefits over multiport laparoscopy surgery (MPS) including the incision being virtually “scarless” as it is hidden within the umbilicus, reducing tissue trauma, leading to lower postoperative pain, faster recovery, shorter hospitalization, and a quicker return to normal activities [4]. However, the technical challenges of LESS can pose difficulties for inexperienced laparoscopic surgeons during the early stages of LESS practice, such as the loss of the triangle structure formed between instruments in MPS, combined with the restricted freedom of instrument movement and potential instrument interference through the small skin incision of the LESS port system.

To date, there is no standardize on how many LESS procedures a surgeon needs to pass, or studies measuring the learning and teaching performance on LESS skills are even lacking. The cumulative sum (CUSUM) learning curve [5], a statistical method that assesses a surgeon’s experience based on the mean value of surgical parameters, including operation time and blood loss, is one efficient way to evaluate a surgeon’s performance [6, 7]. However, for individual surgeons, hands-on learning and detailed surgical skills taught by experienced doctors are more meaningful, and this can only be achieved through ample first-hand experience, both surgeon and assistant’s posture, muscular tension, shoulder/arm/wrist angle and hand movements are essential in the LESS approach. The word “ergonomics” originates from the Greek word “ergon” meaning work and “nomos” meaning law. It refers to the science of designing work environments that promote efficiency, safety, and comfort in the “human-technology-environment” system [8]. In surgery, ergonomics is used to assess the muscle and wrist joint activity of the surgeon to reduce their workload. The recommended ergonomic position for LESS is based on anatomy and physiology to ensure comfort and efficiency during the procedure, thus shorten the operation time, reducing bleeding and injuries, as well as benefiting patients. Surgeon A, as the first author in this article, reviewed her learning curve on LESS produce, use the concept of ergonomics to focus on the self-guided performance of educators, teach surgeon B to perform LESS surgery, aiming to benefit more surgeons, not just those in the field of gynecology.

## Methods

### Patient data collection and postoperative outcomes

This study involved 619 patients who performed LESS surgery for benign gynecological pathologies at Shanghai General Hospital affiliated to Shanghai Jiao Tong University School of Medicine, between January 2021 and April 2022. The study was approved by the institutional review board (IRB) at Shanghai General Hospital, Shanghai Jiao Tong University School of Medicine (2022KY061), and all patients provided informed consent for their medical information to be used for research purposes. Patient demographics and perioperative information were collected from the electronic hospital medical information system software (HAITAI, version 3.0, Nanjing, China) from the time of surgery until hospital discharge.

### Inclusion or exclusion criteria

Patients diagnosed with ICD-11 codes listed in supplementary Tables 1, who performed LESS approach procedures such as salpingectomy, ovarian cystectomy, adnexectomy and myomectomy, were selected for the study. The exclusion criteria for LESS procedures were the same as those for minimally invasive surgery, including serious adhesion and organ failures such as cardiovascular, respiratory, urinary, or nervous system failure.

### Surgical instruments

The surgical instruments used in the procedure included a single-hole multichannel port with two 5-12 mm and two 3-5 mm channels (from Beijing HangTian KaDi Technology R&D Institute, Beijing, China) and made of silicone material which increased the angle of the operation channel, improved the freedom of movement of the instruments, maintain gas tightness to avoid gas leakage as well as smoke interfering with the vision. Conventional transperitoneal laparoscopic surgical tools include: a 10 mm diameter 30-degree laparoscope lens (from Olympus Surgical, Japan or Storz HD, Germany), dissecting forceps, laparoscopic rod, non-traumatic forceps, needle-holding forceps, laparoscopic scissors, suction and irrigation tubes, a 5 mm single-toothed tenaculum, unipolar hook, bipolar grasper (from KANG JI Medical Equipment Co., Ltd, Hangzhou, China), monopolar scissors (from Johnson & Johnson (China) Medical Equipment Co., Ltd), and a 37 mm/45mm V-LocTM absorbable wound closure device (from Covidien, USA).

### Statistical methods

Data was presented as mean ± standard deviation (x ± s) with 95% confidence intervals (95% CI). Statistical significance was set at P < 0.05. Demographic variables were compared between groups using chi-squared or Fisher’s exact test. Perioperative data was analyzed using Student’s t-test or single-factor ANOVA for continuous variables, and Mann-Whitney test for non-parametric variables. All analysis was conducted using SPSS Statistical Software Version 25.0 and graphed using GraphPad Prism Software Version 9.0.

## Result

### Gynecologists’ learning curve

Surgeons enrolled in the research were identified by unique numbers A and B. Surgeon A had 10 years of experience in MPS with 6 years as an assistant surgeon and 4 years as an independent first surgeon performing simple to complex gynecological procedures. From January 2021 to April 2022, Surgeon A performed 580 LESS procedures with a 100% success rate, including salpingectomy (268 cases), ovarian cystectomy (205 cases), adnexectomy (33 cases), and myomectomy (74 cases). 5 myomectomy cases in January 2021 with an additional port were not included in the study as they were considered reduced-port laparoscopy with a different operating space.

Surgeon B had 3 years of experience as an assistant surgeon in MPS and limited experience as a first surgeon in simple CLS procedures such as salpingectomies and ovarian cystectomies. Surgeon B performed a warm-up task and watched a step-by-step video of the procedure before beginning LESS in January 2022, performing salpingectomies (19 cases) and ovarian cystectomies (20 cases) under the guidance of Surgeon A.

The cut-off points chosen for three phases in surgeon’s learning curve depend on the number of LESS surgeries performed, the difficulty level of surgery and surgeon’s proficiency. The first stage being novice, the second stage intermediate, and the third stage routine. The difficulty of surgical skills including salpingectomy as simple, ovarian cystectomy and adnexectomy as general, and myomectomy as medium difficulty.

### Surgical approach

#### Approach A

**Fig. 1:**
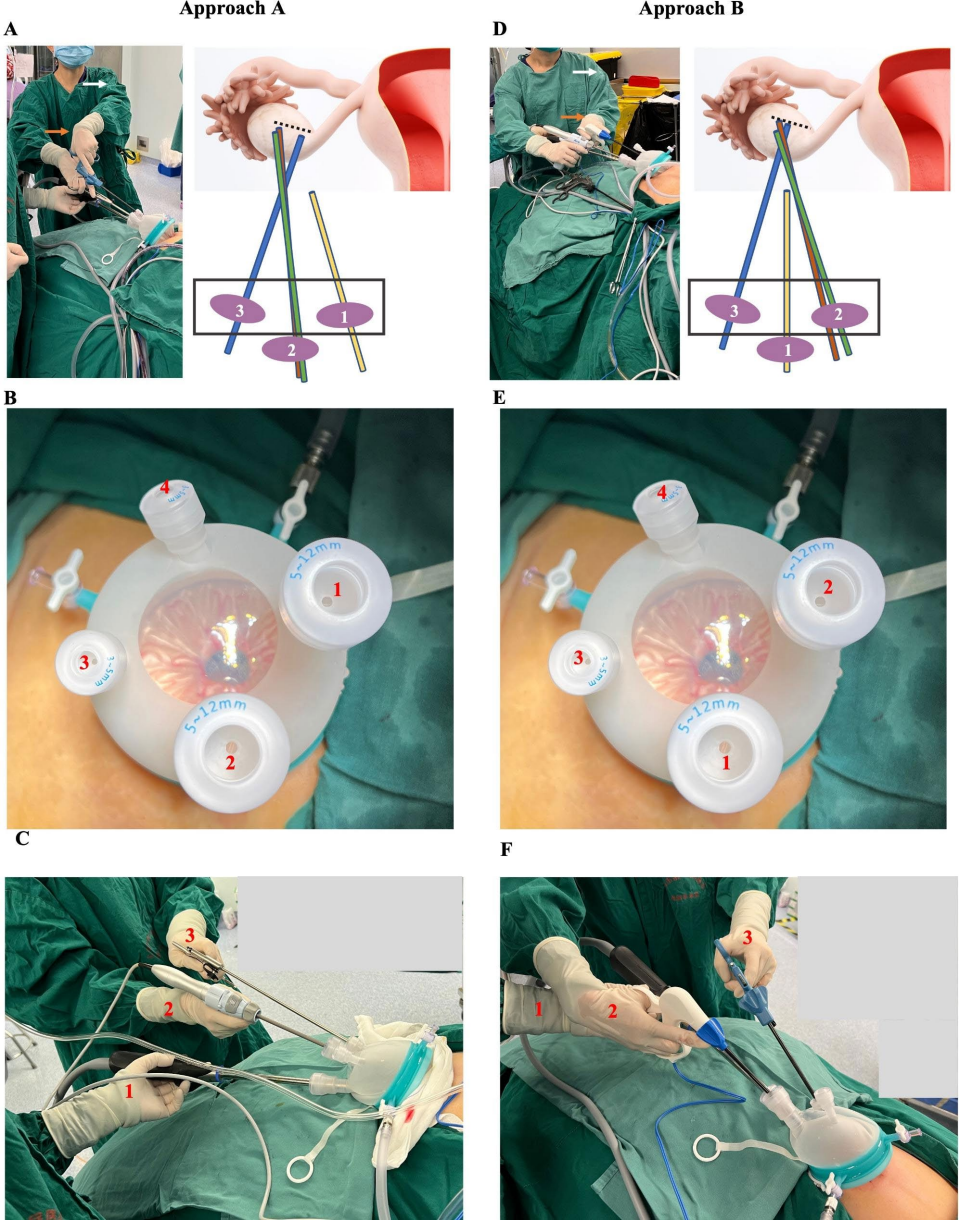
Schematic diagram of LESS surgical approaches A and B. Surgical Approach A: **A.** The instruments (channels 2, 3, and 4) are all placed on the left side of the video laparoscope (channel 1) through a single-hole cannula trocar device (**B.** The first surgeon kept the strength and angle of the shoulders (white arrow) and arms to help the wrists (orange arrow) and fingers adjust the operating position. **C.** The first surgeon’s right hand should remain tight and powerful to hold the instrument to grasp the target tissue. Surgical Approach B: **D.** The instruments (channels 1, 3, 4) are placed on both sides of the video laparoscope (channel 2) through a single-hole cannula trocar device (**E.**); The first surgeon should form a sufficient cross triangle angle with both hands (white arrow). **F.** The first surgeon should open arms and hands (white arrow) to perform the operation to obtain more space. **C. F.** Marker No. 1–3 on the hand represents the corresponding operation channel on the single-hole cannula trocar device. Instruments in **A**. and **B**: 30-degree video laparoscope (yellow), dissecting forceps (blue), monopolar scissors (green), channel (purple round), trocar device (black frame)

The primary surgeon standing on the left and the assistant holding the video-laparoscope on the right. All instruments are placed through one 5–12 mm and 3–5 mm channel on the left side of the video laparoscope through another 5–12 mm channel of the single-hole cannula trocar device (Fig. 1). The bipolar grasper or monopolar scissors are usually placed through channel 2, held by the main surgeon’s right hand, and dissecting forceps through channel 3 or 4, held by the main surgeon’s left hand (Fig. 1A, B, C). This approach was chosen by Surgeon A because of her height of 152 cm and to avoid discomfort with crossing and touching the assistant doctor’s hand.

#### Approach B

The instruments (channels 1, 3, and 4) are placed on both sides of the video laparoscope (channel 2) separately through a single-hole cannula trocar device (Fig. 1D, E, F). In this method, the right hand of the main surgeon has to ride over the assistant doctor’s hand who holds the laparoscope lens, sometimes causing a cross and touch. Most surgeons, including Surgeon B, chose this method, but they needed to lift their hands during the operation. If the height of the first surgeon was over 165 cm, this position did not bring much difficulty.

**Fig. 2:**
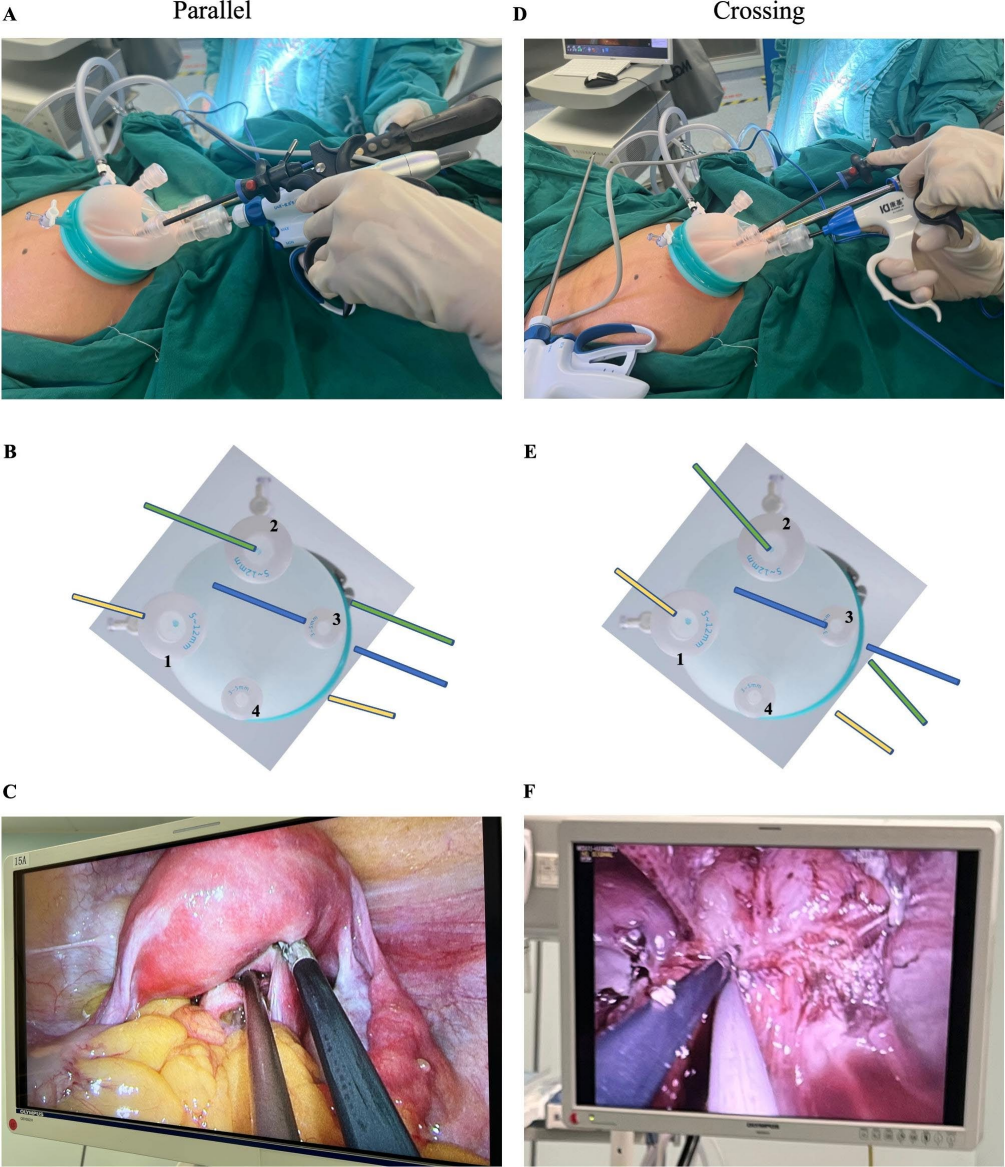
Parallel and crossing technology in LESS. **A.B.C.** In parallel technology, there were the narrow angle between the instruments, the surgeon dominant the instruments along longitudinal depth of the operation field; **D.E.F.** For crossing technique, the instruments were crossed at the same plane and moved in the opposite direction. Instruments in **B**. and **E.**: 30-degree video laparoscope (yellow), dissecting forceps (blue), monopolar scissors (green)

Both parallel and crossing technology was alternately applied to establish triangulation space in LESS [9]. Parallel technology involves a narrow angle between the instruments with the surgeon dominating the instruments along the longitudinal depth of the operation field. (Fig. 2A, B, C); Crossing technique involves crossing the instruments at the same plane and moving in the opposite direction, which forms good tension during the operation (Fig. 2D, E, F).

### LESS device choice

### Ergonomic element of the LESS technique

#### The main surgeon skills

For beginner surgeons, lengthening instruments and integrated laparoscope lens could be used, while for experienced surgeons, MPS surgical instruments and lens were also applicable. Lubricating oil could be used on the instruments and lens to improve instrument jamming.The key to success in LESS surgery is to have good coordination of location, orientation, movements, functions, and flexibility of the shoulders, arms, elbow, wrist, and finger joints, and to maintain strength, tension, ambidexterity, depth perception, and repetition of muscular efforts while controlling the instruments.

**Fig. 3:**
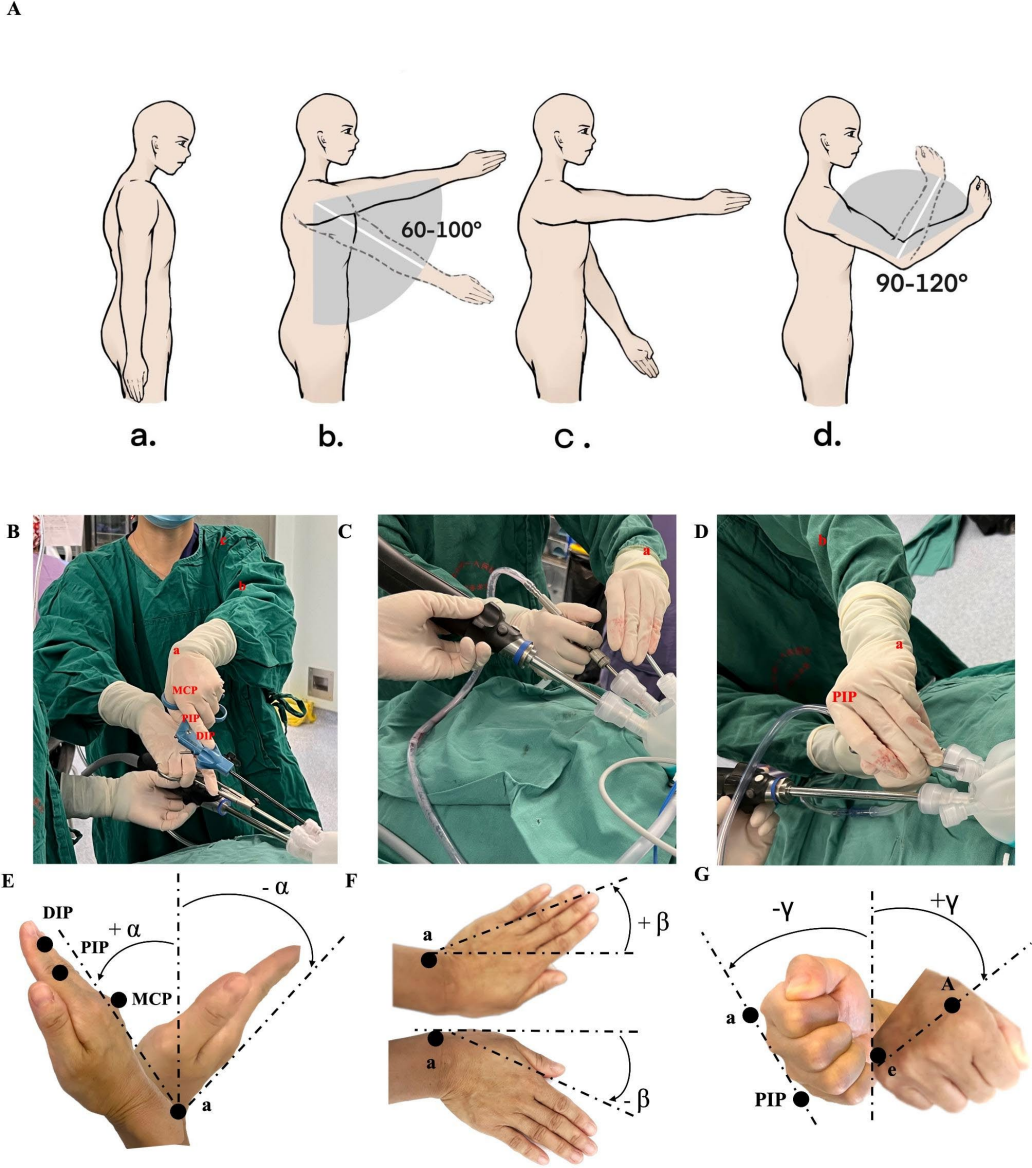
Ergonomic element of the LESS technique. **a** The surgeon stands symmetrically with the straight back and slightly forward neck to maintain equilibrium. **b.** Keep shoulders in an unraised, neutral anatomic position, raised from 60° to 100° with upper arm straightly. **c.** Right arm usually elevated higher than left arm, or in some situation both arms crossed in order to maintain the position. **d.** The elbow joint is flexed to form an angle of 90° to 120° between the upper arm and forearm of the main surgeon. **B.E.** Flexion and extension of the wrist is measured as the positive or negative angle α, separately, between the humeroradial and neutral position lines. The humeroradial line was defined by markers a on the trapezium bone of the wrist and b on the knuckle of the index finger. The neutral position line starts with marker a located on the humeroradial bone of the elbow. Shoulder abduction along the biceps muscle was pointed out with marker c. **C.F.** Radial and ulnar deviation were also measured by markers a and b, and the positive angle β represented the radial deviation, while the negative angle β represented the ulnar deviation. **D.G.** The protonation with positive angle γ and supination with negative angle γ of the forearm is measured with marker a on the knuckles of the index finger and marker c on the little finger

1. The surgeon maintains proper posture with straight back and slightly forward neck (Fig. 3A-a).

2. Keeping the shoulders in a neutral and unraised position, the height of the raised arm ranging from 60° to 100° depending on the surgeon’s height (Fig. 3A-b, B).
3. Right arm is usually elevated higher than the left arm, or both arms be crossed (Fig. 3A-c).
4. The main axis of the shoulders, arms, and elbow should be vertically aligned, with the elbow joint flexed to form an angle between 90° and 120°(Fig. 3A-d).
5. The movements of the wrist and finger joints are maintained by protonation and supination of the metacarpophalangeal joint (MCP), distal interphalangeal joint (DIP), proximal interphalangeal joint (PIP), and flexor digitorum profundus (FDP) and flexor digitorum superficialis (FDS) (Fig. 3B). The wrist moves between flexion and extension, from 0° to 30° (Fig. 3C, D), smoothly and with positive or negative angle α and β of radial and ulnar deviation (Fig. 3E, F). The rotation of the fist (angle γ) can also be adjusted to suit the operating position (Fig. 3G) [10, 11].
6. Various hand functions interchanged including tip/key/pulp pinch, power grasp, flexion or extension with thumb and each finger [12].

#### The assistant surgeon skills

Although the assistant surgeon may not perform any surgical actions, their proficiency in the following skills are necessary for beginners to adapt the operating environment.

1. Familiarity with the instruments, anatomy, surgical procedures, and the habits of the main surgeon.
2. Achieving hand-eye synchronization, ensure the clear and stability of the laparoscope lens during the procedure.
3. The assistant needs to be quick and flexible to adjust the surgical field to obtain a complete image. In case of collision between the video-laparoscope and the instruments, timely back up the lens and provide priority space for the surgical device. Bring the video-laparoscope closer or farer to the surgical field according to closer observation or overall view.
4. Adjust the direction and angle of 30° surface of the lens to maintain more comprehensive image.

### Hands-on performance of detailed operation

#### Salpingectomy

**Learning Point**:

**1. Ensure optimal spatial awareness and hand-eye coordination.**

**2. Modify the placement and alignment of instruments through limb angular movements.**

**Fig. 4:**
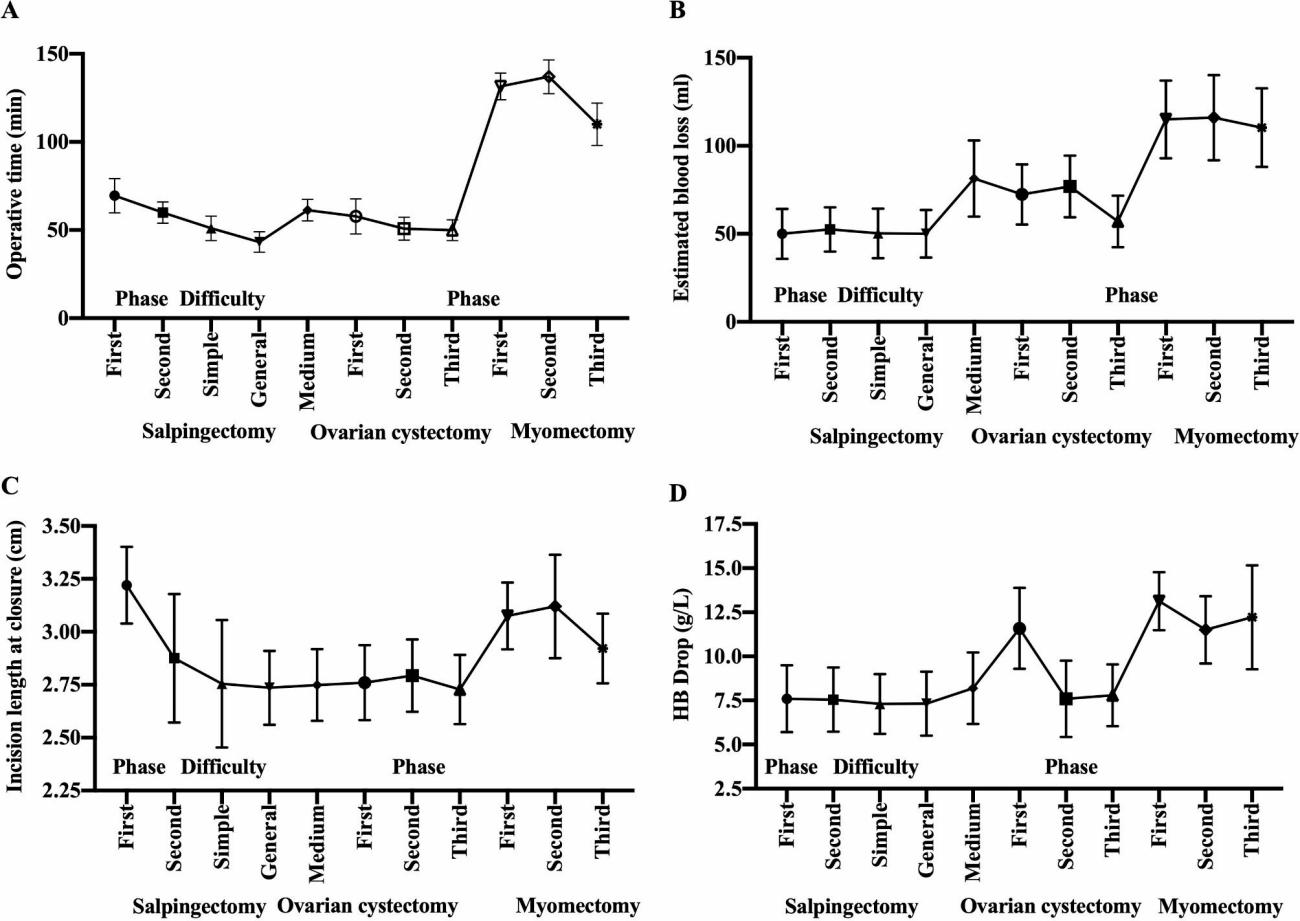
Perioperative data between different groups in salpingectomy, ovarian cystectomy and myomectomy conducted by surgeon A. (**A**) Operative time (min) (**B**) Estimated blood loss (ml) (**C**) Incision length closure (cm) (**D**) HB drop (g/L) among different learning periods and difficulty levels in salpingectomy, ovarian cystectomy and myomectomy

Surgeon A performed 268 cases of salpingectomies under LESS, her proficiency gradually increases with novice (20 cases), intermediate-level (20 cases), and routine (remaining cases). In the first phase, the main challenges were the interference of instruments leading to difficulty in grasping or coagulating. To address these issues, she increased the transumbilical incision length to 3.0-3.5 cm to ensure ample operating space. Through practice and paid attention to the above ergonomics learning points, she conquered instrument collisions and the use of cross-hand techniques with wrist and elbow movements, and gradually reduced the incision length to 2.5–3.3 cm. Surgeon A’s prior experience with MPS aided her in forming a good laparoscopic spatial sensation. The results showed a significant difference in operative time (min), fluid infusion (L) and incision length at closure between the novice and intermediate-level groups, possibly related to surgical proficiency. (Supplementary Tables 3, Fig. 4A-D) (P ≤ 0.05).

The remaining cases of tubal pregnancy and other cases of salpingectomies were grouped depending on the level of difficult technology: 113 cases of tubal pregnancy were simple, 36 cases of cyst of fallopian tube and 5 cases of torsion of fallopian tube were general, 41 cases of abscess of salpingitis and oophoritis were medium. With increased LESS technical difficulty, although obvious significant differences in operative time, fluid infusion, estimated blood loss (ml), and postoperative HB and HB drops (g/L) were observed (Supplementary Table 3) (Fig. 4A-D), no significant differences between the three groups for incision length at closure (P > 0.05) were observed, which demonstrated that surgeon A could adapt the smaller TS-LESS incision.

#### Ovarian cystectomy and adnexectomy

**Learning Point**

**1. Proficient in blunt separating, coagulation, cutting and handling produce.**

**2. Improve basic LESS technique: grasp, lift, transfer and place objects.**

**Fig. 5:**
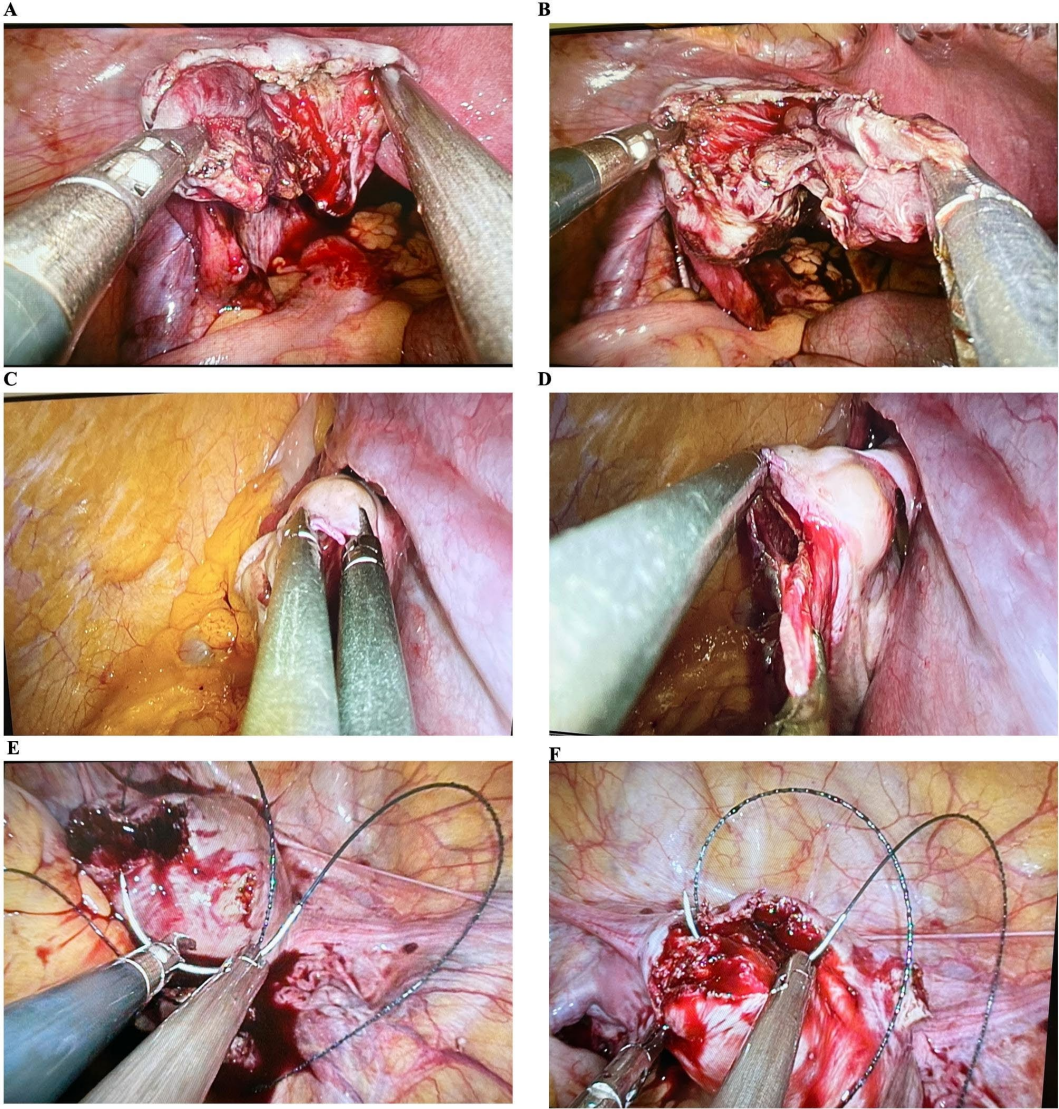
LESS surgical skills in ovarian cystectomy and myomectomy. **A.B.** Grasp the edge of the ovarian cortex by the left hand, seek a suitable angle to suture, or coagulate the remaining ovarian cortex by the right hand. Blunt separating the cyst from the ovarian cortex precisely with proper power. **C.D.** Ovarian cystectomy on patient with cystic teratoma of ovary accompanied with pregnancy at 18 gestational weeks. **C.** The ovarian cyst was grasped with two dissecting forceps, and the first surgeon should overcome the “chopstick effect”. **D.** Separation of the cystic cortex from the normal ovary by two dissecting forceps must be done very gently to avoid touching the uterus. **E.F**. Myomectomy of a patient with uterine leiomyoma. **E.** Surgeon A held the needle with the needle holder by the right hand and dissecting forceps with the left hand to keep the right position. The needle holder should be placed 2/3 near the end of the needle at a 90-degree angle to the needle. **F.** When suturing, the needle holder with the needle entered from one edge of the retracting myometrium to the opposite side, and the dissecting forceps clamped the opposite side of the incision edge to fix the uterus

A total of 205 ovarian cystectomies were divided into three groups based on surgical difficulty: 59 cases of corpus luteum hemorrhage or rupture were classified as simple; 76 cases, including follicular cyst, cystic ovary torsion, cystic teratoma, ovarian fibroma, ovarian serous cystadenoma, and ovarian pregnancy, were classified as general. Seventy cases of endometrial cystoma of the ovary and its rupture were classified as medium. During the novice period, Surgeon A found it difficult to handle smooth, round ovarian cysts and struggled to make incisions with scissors or monopolar scissors. The ovarian cortex was also often too fragile to be torn by separation forceps. Since un-proficient skills prolong the operation time, significant differences among three groups divided by surgical difficult were observed in the operative time, fluid infusion, estimated blood loss, HB drop and stay of hospitalization (Supplementary Table 4). However, through exploring the appropriate angle and strength, Surgeon A improved her skills in grasping, lifting, transferring, and placing objects. She became proficient in blunt separation, coagulation, and cutting, consistently handling tissue precisely to avoid injury (Fig. 5A, B), There were no significant differences among the groups for major perioperative parameters in adnexectomy (Supplementary Table 5), thus resulting in patients’ faster recovery.

In March 2022, Surgeon A successfully performed two cases of cystic teratoma of the ovary accompanied by pregnancy at 17 and 18 weeks of gestation. The instruments were passed through the incision port and the removal of the ovarian cysts was performed gently and accurately to avoid touching the uterus (Fig. 5C, D). To ensure safety, a single-site skin incision 2 cm above the umbilicus was made due to the proximity of the uterus to the umbilicus (Supplementary Table 4). These two cases verified the feasibility of LESS during pregnancy and provided valuable experience for the surgeon.

#### Myomectomy

**Learning Point**

**1. Obtain good power and angle with instruments, well with shoulder, arms, wrists and fingers.**

**2. Ambidexterity, depth perception, continuous postures, forces, and repetition with muscular effort.**

Surgeon A performed a learning process for conducting 74 cases of uterine leiomyoma with LESS which could be divided into three phases: first (n = 8), second (n = 10) and third (n = 56). During this process, several factors like myoma diameter, location, multiple large fibroids, previous surgeries, etc. posed challenges and increased the risk of intraoperative blood loss. The size of the myoma, operational time and incision length at closure was significant among three learning stage (P ≤ 0.05) (Supplementary Table 5) (Fig. 4A-D), a section of the intravenous infusion tube could be used in the 3–5 mm channel to discharge smoke produced by energy instruments. In the initial stages, it was difficult for her to control the traction and countertraction force for dissecting the myoma, but she improved her skills over time by adjusting her arm, wrist, and finger movements.

After removal, the myometrium was restored with a V-LocTM absorbable wound closure device by continuously suturing in two layers. In this step, surgeon A faced difficulties in adjusting the needle angle and avoiding interference with surgical instruments under a crowded space, she applied wrist and elbow angular movements in the flexion-extension plane from 0° to 180° as fully extended, along with elbow joint flexed 110°–120° between the upper arm and forearm. When suturing, she held 2/3 near the end of the needle with appropriate amount of force for accurate fixation, insertion and withdraw to adopt the proper angle. The needle was entered from one edge of the retracting myometrium to another side, and the dissecting forceps clamped the opposite edge of the myometrium to fix the uterus (Fig. 5E, F). Therefore, the operation time, fluid infusion, estimated blood loss and incision length at closure were significantly different among three learning phases (Supplementary Table 5).

### Learning experience for surgeon B under the direction of surgeon A

Surgeon B, who has limited experience in CLS, found it challenging to adopt the “chopstick effect” in LESS. In the first period, she performed 5 cases of salpingectomies and 5 cases of ovarian cystectomies, and in the second period, she performed 14 cases of salpingectomies and 15 cases of ovarian cystectomies (Supplementary Table 6). Since the main difficulty was due to the cramped space, surgeon B faced difficulties in grasping the tissue and finding the right angle to fix the ovarian cyst in the beginning. Surgeon A helped surgeon B in holding and transferring instruments, taking advantage of the soft and flexible direction of the multichannel trocar, adjusting the position of the instrument to the video-laparoscope to get the best operating angle, and maintaining strength with both hands to grasp the target tissue. As surgeon B conducted more cases, she significantly increased her technical proficiency. Surgeon A pointed out the obstacle mainly on the ergonomic aspect that surgeon B or most initial learners encounter in the next paragraph.

### Assessment for LESS skills in three learning periods on surgeons A and B

Surgeons A and B’s LESS skills were evaluated through different Modified from the objective structured assessment of technical skills (OSATS) [13, 14], the evaluation consisted of ten aspects such as scope handling, instrument usage, laparoscopic spatial sensation and view field, equipment selection, flow of procedure, blunt separation, coagulation/cutting, tissue handling ability, coordination with instruments, and communication with the assistant (Table 1).

**Table 1:** Assessment form for hands-on performance of LESS skills in three learning periods on Surgeons A and B

Score	Assessment	Surgeon A			Surgeon B	
Phase		First	Second	Third	First	Second
(1) Basic knowledge of anatomy in pelvic						
3	Fairly familiarity with anatomy in pelvic even on occasion of server adhesion	3	3	3		
2	Knew well anatomy in pelvic but on occasion of server adhesion could not distinguish				2	2
1	Insufficient knowledge of anatomy in pelvic					
(2) Knowledge of surgical procedure						
3	Demonstrated familiarity with all steps of procedure	3	3	3		
2	Knew all important steps of procedure, did not have to be stooped at any nodal points					2
1	Insufficient knowledge looked unsure and hesitant, had to be stopped at one or more nodal points				1	
(3) Scope handling and instruments usage						
3	Proficient uses instruments, no stiffness or awkwardness		3	3		
2	Competent use of instruments but occasionally appeared stiff or awkward	2				2
1	Repeatedly makes awkward or tentative moves with instruments through inappropriate use				1	
(4) Laparoscopy spatial sensation and view field						
3	Maintain good laparoscopic spatial sensation and clear view of laparoscopy field					
2	Maintain clear view but occasionally lost or did not focus on the main instruments		3	3		2
1	Not able to achieve clear laparoscopy view, requiring take over	2			1	
(5) Chose suitable equipment						
3	Clearly, understanding equipment setup and chooses appropriate equipment for intervention		3	3		
2	Required prompting for proper preparation of equipment	2				2
1	Unable to properly prepare and choose appropriate equipment for intervention				1	
(6) Blunt separating, coagulation or cutting produces alternately						
3	Proficient in blunt separating, coagulation or cutting produces alternately		3	3		
2	Generally, proficient in blunt separating, coagulation or cutting produces	2				2
1	Poor coordination with Blunt separating, coagulation or cutting produces				1	
(7) Precisely tissue handling ability and avoid tissue injuring						
3	Consistently handled target tissue precisely with proper power and completely avoid tissue injure	3	3	3		
2	Careful handling of tissue but occasionally failed to catch or hold, occasionally cause inadvertent damage but not important organ like bladder, intestinal or urinary.					2
1	Frequently use unnecessary force on tissue, and unnecessary cause damage to the target tissue and resulted in bleeding but not important organ like bladder, intestinal or urinary.				1	
(8) Coordination with instrument with shoulder, arms, wrists and fingers						
3	Obtain good power and angle with instruments, coordination well with shoulder, arms, wrists and fingers		3	3		
2	Generally, obtain power and angle with instruments and hands but need improvement	2				2
1	Poor coordination with instrument by hand				1	
(9) Postures, forces, and repetition with muscular effort on shoulder, arms, wrists and fingers						
3	Ambidexterity, depth perception, continuous postures, forces, and repetition with muscular effort on shoulder, arms, wrists and fingers		3	3		
2	Generally, obtain postures, forces, and repetition with muscular effort	2				2
1	Poorly obtain postures, forces, and repetition with muscular effort				1	
(10) Communication with assistant						
3	Clear instruction and cooperation with assistant and achieve excellent teamwork		3	3		3
2	Reasonable cooperation with assistant but occasional face obstacle on communication	2			2	
1	Poor communication with assistant, and achieve teamwork with disappointment					
Total		23	30	30	12	21
Overall performance						
3	Competent		3	3		
2	Reasonably competent but requires further training	2				2
1	Adequate and requires continued basic training				1	

For surgeon A, benefitting from fairly familiarity with anatomy in the pelvis and all steps of operation, which is similar in MPS, shortly after the novice period, she became proficient in the remaining aspects in scope handling and instrument usage, laparoscopic spatial sensation and view field, chose suitable equipment, flow of procedure, blunt separating, coagulation or cutting produces alternately, precise tissue handling ability and avoided tissue injuring, coordination with instrument with shoulder, arms, wrists and fingers, postures, forces, and repetition with muscular effort and communication with assistant.

Surgeon A scored 23 in the first phase and improved to 30 in the second to third phases, while Surgeon B scored 12 in the first phase, but improved to 21 in the next phase with guidance from Surgeon A. In addition, surgeon A told B, “May be I am your senior doctor, you felt timid to pose your right across my hand which hold the video-laparoscope; You should be confident to act as the main surgeon to open arms to operate to obtain more space, and suppose I am just an assistant doctor.” After detailed explanation and guidance under surgeon A, in the subsequent 15 LESS procedures, Surgeon B was advised by Surgeon A to be confident and act as the main surgeon to get more space. After receiving guidance, Surgeon B improved her operating angle and force and became more confident in her LESS procedures.

## Discussion

There are no absolute contraindications for LESS today, while a large uterus size with multiple myomas, previous abdominal surgeries or obesity remains a relative contraindication at the beginning of LESS [15]. Evidence underlined that myoma size > 12 cm, number ≥ 5 myomas, location as intramural myomas between 8 and 12 cm diameter [16–18], women with a BMI greater than 30 can also have prolonged operation times. It is suggested that a surgeon needs to perform 30–55 cases of benign gynecological operations to become proficient in the LESS technique [19]. The CUSUM learning curve indicates that the degree of mastery in the LESS technique is dependent on factors such as adhesion grade, surgical type, and surgeon experience. The operation time in the second learning phase (last 6 cases) was lower than the first learning phase (initial 6 cases). The complication rate for LESS in benign gynecological diseases ranges from 0.29 to 1.4% [6, 20]. Adequate training is crucial to minimize operating time and decrease the occurrence of complications [21, 22]. The frequency of overall postoperative complications and the rate of conversion to laparotomy decrease as the surgeon’s experience increases [23], and result in a two-fold increased risk of bladder injury and a four-fold higher risk of ureter damage [22]. Gynecologists with a background in abdominal, vaginal, and MPS techniques are better prepared for LESS as basic surgical steps, laparoscopic spatial sensation, and familiarity with anatomy are consistent.

The most valuable learning experience comes from individual surgeons through practical operations, which cannot be quantified by data. The hands-on performance learning curve for LESS in benign gynecological diseases follows a gradual stepwise process. The procedure is accompanied by challenges for the surgeon, including musculoskeletal problems, difficulty with traction and triangulation, mental fatigue, irritability, exhaustion, instrument manipulation, and the use of cross-hand techniques with wrist and elbow movements [24]. Previous ergonomic studies mostly concentrate on preventing musculoskeletal injury, physical burdens in endoscopy, laparoscopy [25] or robotic surgical [11]. Annie E [26] use inertial body sensors to measure joint angles to evaluate the ergonomics of surgeons during endoscopic and microscopic otologic surgeries. Our study builds upon these concepts, using the difficulty level of the operation as a starting point, and progressing from salpingectomy to ovarian cystectomy and myomectomy. Our hands-on approach highlights the specific details of how a surgeon should perform a particular procedure, as well as the method a senior surgeon used to teach a junior surgeon.

In the novice period, surgeon A honed her skills in adjusting her limb movements to properly locate and orient herself during LESS laparoscopic procedures. She also developed a strong spatial sense and became proficient in setting up equipment and selecting the right tools for the operation. Due to the limited space within the single-port device and the need for triangulation and visualization, she had to adopt a greater wrist deviation and range of motion in her movements. As she progressed to intermediate level, she mastered the finer points of the procedure, including grasping and suturing tissue, transferring objects, and controlling bleeding with different instruments. By the time she reached the routine stage, she had achieved ambidexterity, depth perception, and the ability to maintain fluid and repetitive movements with minimal effort. This allowed her to effortlessly control the instruments and complete the procedure with ease, leading to shorter operating times, reduced perioperative bleeding, and improved patient outcomes. Through step-by-step training and hands-on experience, she became more proficient and specialized, thus reducing operating time and perioperative bleeding and improving patient outcomes.

Providing effective surgical training and support during hands-on operations can be a challenge for young surgeons as resources for acquiring advanced LESS skills are limited. Surgeon B struggled to form a proper cross-triangle angle with her arms and hands, and lacked the strength to hold the instrument correctly. To help her improve, Surgeon A acted as a mentor, pointing out the common difficulties faced by novice LESS surgeons and demonstrating the ergonomic principles required for success. To overcome the limitations imposed by the narrow entrance of LESS port systems and prevent surgical instruments from interfering with each other, with practice and guidance, surgeon B was able to develop the proper power and angle with her instruments and improve her hand-eye coordination, the key to success in LESS surgery is to have good coordination of location, orientation, movements, functions, and flexibility of the shoulders, arms, elbow, wrist, and finger joints, and to maintain strength, tension, ambidexterity, depth perception, and repetition of muscular efforts while controlling the instruments. Recently, LESS was reported to conducted in patients with early-stage type I endometrial cancer [27], stage 1B1 cervical adenocarcinoma desiring future fertility [28], or nephroureterectomy [29], which improved the surgical out-come and QoL of patients with reliability, effectiveness and safety.

## Limitation

The limitations of this study should be acknowledged, as its retrospective nature and single institutional setting. In addition, surgeon A introduced her own learning and teaching experiences on LESS, while lacks a reasonable sample size which may limit its generalizability. We would explore our study to additional surgeons in their learning curve to improve the validity of the research. Also in order to improve the validity of our findings, future research should consider incorporating objective measurement techniques such as 3D motion analysis and the use of inertial measurement unit sensors [30]. A laparoscopic simulator like an intracorporeal suture could also be used to evaluate and validate the canine ergonomic abdominal simulator for the acquisition of basic laparoscopic skills, reflected in the surgical performance scores [31]. These would provide quantifiable data on kinematic variables and detailed ergonomic methodologies.

## Conclusion

This study highlights the challenges faced by gynecologic surgeons with limited experience in LESS surgery as they attempt to adopt it for the treatment of benign gynecologic conditions. The study emphasizes the importance of ergonomics in surgical procedures and its role in reducing musculoskeletal discomfort, which has not been extensively reported previously. The authors hope that their ergonomic hands-on performance learning and teaching methodology will be incorporated into the evaluation criteria for LESS procedures.

### Electronic supplementary material

Below is the link to the electronic supplementary material.

Supplementary Material 1

## Data Availability

The datasets used and analysed during the current study are available in the Supplementary Information files.

## Abbreviations

3D: Three-dimensional
BMI: Body mass index
CLS: Conventional laparoscopic surgery
CM: Centimeter
DIP: Distal interphalangeal joint
EMG: Electromyographic
FDP: Flexor digitorum profundus
FDS: Flexor digitorum superficialis
HB: Hemoglobin
HR: Hazard risk
ICD-11: International Classification of Disease, Eleventh Revision, Clinical Modification (ICD-11)
LESS: Laparo-endoscopic single-site
LM: Laparoscopic myomectomy
mAFS: Modified adhesion classification standard
MCP: Metacarpophalangeal joint
MPS: Multiport laparoscopic surgery
OSATS: Objective Structured Assessment of Technical Skills
PID: Pelvic inflammatory disease
PIP: Proximal interphalangeal joint
sEMG: Surface electromyography
TOA: Tub-ovarian abscess
Tu-LESS: Transumbilical laparoendoscopic single-site
