# Supplementary material for: Ergonomic learning curves on gynecological laparoendoscopic single-site (LESS) surgery
Source: BMC Surg. 2023 Oct 27;23:327. doi: 10.1186/s12893-023-02241-x (PMC10612358; doi:10.1186/s12893-023-02241-x)
Supplement: Supplementary file 1 — Supplementary Material 1 [file 12893_2023_2241_MOESM1_ESM.docx]

**Supplementary Table 1.** International Classification of Disease, Eleventh Revision, Clinical Modification (ICD-11) codes

| ICD-11 | Diagnosis | Description |
| --- | --- | --- |
| GA05.3 | Abscess of Salpingitis and oophoritis | End-stage process of acute PID, marked by pelvic mass palpable during bimanual examination. Usually bilateral but can be unilateral |
| GA10.2 | Superficial endometriosis of ovary | Chocolate cyst of ovary  Endometrial cystoma of ovary  Endometriosis on the ovarian cortex, containing typical or subtle lesions, |
| GA10.3 | Deep endometriosis of ovary | Ectopic growth and function of endometrial tissue extending more than 5 millimetres under the peritoneal surface, associated with remaining vestigial tissue from the wolffian or mullerian duct, or fragments of endometrium refluxed backward into the peritoneal cavity during menstruation, or may be idiopathic. |
| GA11 | Adenomyosis | A condition of the uterus characterised by endometrial tissue growth in the myometrium, hypertrophy of the myometrium, and heavy or prolonged menstrual bleeding, dysmenorrhoea, dyspareunia, bleeding between menstruation, infertility, or is asymptomatic. |
| GA17.Y | Of tubal origin | Tubal: block, occlusion, stenosis  Use additional code for any associated peritubal adhesions |
| GA17.0 | Cyst or polyp of fallopian tube | Cyst located on the fallopian tube at the outside of the fimbrial end |
| GA18.0 | Follicular cyst of ovary | Cyst of graafian follicle  A condition of the ovary, caused by a follicular growth or involution due to failure of ovulation. This condition is characterized by the non-neoplastic formation of closed sac-like structures filled with fluid on or in the ovary and lined by layers of granulosa cells. |
| GA18.Y | Other noninflammatory disorders of ovary, fallopian tube, and broad ligament | Rupture of ovary or fallopian tube  Hematosalpinx of ovary or fallopian tube |
| GA18.1 | Corpus luteum hemorrhage or rupture | A condition affecting females, caused by the expansion of the corpus luteum with air, blood, or fluid in the ovary after ovulation of a follicle. This condition is characterized by a mass of up to 10 cm in diameter. |
| GA18.2 | Theca-lutein cyst of ovary |  |
| GA18.4 | Ovarian endometriosis cyst |  |
| GA18.5 | Torsion of ovary, ovarian pedicle, or fallopian tube | Partial or complete rotation of the ovarian vascular pedicle caused by benign ovarian cyst or ovarian hyperstimulation |
| GA18.6 | Other and unspecified ovarian cyst | Serous cyst of ovary, Simple cystoma of ovary |
| 2F32.Z | Benign neoplasm of ovary |  |
| 2F32.0 | Cystic teratoma of ovary |  |
| 2F32.1 | Ovarian fibroma |  |
| 2F32.3 | Ovarian serous cystadenoma |  |
| 2E86.0 | Uterine leiomyoma, fibromyoma, myoma | Intramural myoma, Subserous myoma,Submucous myoma  A well-circumscribed benign smooth muscle neoplasm characterised by the presence of spindle cells with cigar-shaped nuclei, interlacing fascicles, and a whorled pattern. |
| JA01.Y | Cornual pregnancy |  |
| JA01.Z | Ectopic pregnancy  Tubal pregnancy  Fallopian pregnancy  Rupture of (fallopian) tube due to pregnancy |  |
| JA01.2 | Ovarian pregnancy | A condition characterised by implantation of the embryo within the ovary during pregnancy. |

**Supplementary Table 2.** Clavien–Dindo classification for postoperative complications

| Complication Grade | Description |
| --- | --- |
| Grade I | Any deviation from the normal postoperative course without the need for pharmacological treatment or surgical, endoscopic and radiological interventions. Allowed therapeutic regimens are: drugs as antiemetics, antipyretics, analgetics, diuretics and electrolytes and physiotherapy, wound infections opened at the bedside, transient postoperative fever up to 38.6℃ |
| Grade II | Complications requiring pharmacological treatment with drugs other than such allowed for grade I complications (blood transfusions and total parenteral nutrition are also included) |
| Grade III | Complications requiring surgical, endoscopic or radiological intervention |
| Grade IV | Life-threatening complication (including central nervous system complications) requiring intensive care- management |
| Grade V | death of a patient |

Febrile episodes or unspecified infections is defined as a temperature higher than 38℃ on two occasions at least 12 hours apart included urinary tract, surgical wound, pelvic, chest, port site and vaginal cuff infection or other infection-related conditions. Wound healing complications included hematoma, dehiscence or eventration. Urinary tract complication included urinary retention as inability to empty bladder. Intestinal complications as paralytic ileus and intestinal obstruction. Other complications including pneumonia, myocardial infarction, deep venous thrombosis/pulmonary embolism. Major long-term complications including fistula, pelvi-abdominal pain, urinary dysfunction, bowel dysfunction, pelvic floor condition (prolapse), sexual dysfunction, metabolic disorders, neurological alterations and anesthesia-related alternations.

**Supplementary Table 3**. The patients’ demographic data and surgical outcome in salpingectomy (n=268) performed by surgery A

| Level of Difficult | General | |  | | | Simple | General | | | | Medium |  |  |
| --- | --- | --- | --- | --- | --- | --- | --- | --- | --- | --- | --- | --- | --- |
| ICD-11 | JA01.Z | | | | | JA01.Z | GA17.0 | GA18.5 | GA18.Y | Total | GA05.3 |  |  |
|  | Tubal pregnancy | | | | | Tubal pregnancy | Cyst of fallopian tube | Torsion of fallopian tube | Hematosalpinx of fallopian tube |  | Abscess of Salpingitis and oophoritis |  |  |
| Phase | First | Second | |  |  | Third | First (n=10) Second (n=20) Third (n=54) | | |  | Third |  |  |
| Case | 10 | 20 | | t | p-value | 113 | 36 | 5 | 43 | 84 | 41 | F | p-value |
| Pre-operative | | | | | |  | | | | | | | |
| Age | 32.40±8.82 | 32.70±7.02 | | 0.10 | 0.92 | 33.33±7.38 | 45.25±6.67 | 40.60±3.91 | 51.77±4.11 | 48.31±6.47 | 38.29±6.42 | 113.9 | <0.0001 |
| BMI | 20.46±1.53 | 22.07±2.73 | | 1.73 | 0.10 | 22.02±2.24 | 22.64±2.10 | 21.92±1.88 | 22.69±1.99 | 22.66±2.04 | 22.41±2.17 | 2.202 | 0.1128 |
| HB 1 | 107.2±19.40 | 112.20±15.48 | | 0.77 | 0.45 | 110.10±14.89 | 118.4±11.76 | 123.2±13.07 | 117.2±11.59 | 118.00±11.68 | 115.00±8.24 | 9.34 | 0.0001 |
| Intra-operative | | | | | |  | | | | | | | |
| Operative time | 69.50±9.64 | 59.95±6.03 | | 3.34 | 0.0024 | 51.01±7.00 | 43.94±7.40 | 41.20±4.55 | 42.86±4.24 | 43.23±5.81 | 61.37±6.12 | 111.5 | <0.0001 |
| Fluid infusion | 1.12±0.14 | 0.85±0.12 | | 5.63 | <0.0001 | 0.80±0.14 | 1.08±0.18 | 1.06±0.17 | 1.05±0.17 | 1.07±0.18 | 1.00±0.19 | 72.16 | <0.0001 |
| Estimated blood loss | 50.00±14.14 | 52.50±12.51 | | 0.49 | 0.63 | 50.09±14.05 | 48.89±13.48 | 49.00±14.32 | 51.16±13.84 | 50.06±13.59 | 82.20±21.27 | 74.09 | <0.0001 |
| Incision length at closure | 3.22±0.18 | 2.88±0.30 | | 3.29 | 0.0027 | 2.76±0.30 | 2.78±0.19 | 2.70±0.19 | 2.71±0.16 | 2.74±0.17 | 2.75±0.17 | 0.15 | 0.8594 |
| Post-operative | | | | | |  | | | | | | | |
| HB 2 | 99.60±18.64 | 104.70±15.2 | | 0.79 | 0.43 | 102.80±15.16 | 110.90±11.2 | 116.8±12.70 | 109.8±11.78 | 110.70±11.55 | 106.80±8.25 | 8.93 | 0.0002 |
| HB Drop | 7.60±1.90 | 7.55±1.82 | | 0.07 | 0.94 | 7.30±1.60 | 7.42±1.74 | 6.40±1.52 | 7.35±1.90 | 7.32±1.81 | 8.20±2.03 | 4.14 | 0.0172 |
| Out of bed activity time | 28.20±2.86 | 25.70±3.23 | | 2.07 | 0.05 | 26.94±3.10 | 24.19±3.43 | 23.60±3.21 | 23.72±2.90 | 23.92±3.13 | 26.02±2.59 | 24.25 | <0.0001 |
| Anal exhaust time | 24.80±2.82 | 22.15±3.01 | | 2.32 | 0.0280 | 21.81±2.52 | 26.25±2.72 | 23.40±2.30 | 24.40±3.49 | 25.13±3.24 | 24.73±2.57 | 38.56 | <0.0001 |
| Stay of hospitalization | 2.50±0.53 | 2.40±0.50 | | 0.51 | 0.6170 | 2.49±0.50 | 3.17±0.85 | 3.20±0.45 | 3.14±0.77 | 3.16±0.78 | 4.46±0.50 | 155.9 | <0.0001 |
| VAS | 3.60±1.08 | 3.70±1.22 | | 0.22 | 0.8275 | 3.58±1.12 | 3.11±0.78 | 3.20±0.84 | 2.91±0.87 | 3.01±0.83 | 3.15±0.79 | 8.665 | 0.0002 |

**Supplementary Table 4**. The patients’ demographic data and surgical outcome in ovarian cystectomy (n=205) performed by surgery A

| Level of Difficult: | Simple | General | | | | | | | Medium | | |  |  |
| --- | --- | --- | --- | --- | --- | --- | --- | --- | --- | --- | --- | --- | --- |
| ICD-11 | GA18.1 | GA18.0 | GA18.5 | 2F32.0 | 2F32.1 | 2F32.2 | JA01.2 | Total | GA10.2 | GA18.Y | Total |  |  |
|  | Corpus luteum hemorrhage or rupture | Follicular cyst of ovary | Cystic of ovary torsion | Cystic teratoma of ovary | Ovarian fibroma | Ovarian serous cystadenoma | Ovarian pregnancy |  | Endometrial cystoma of ovary | Endometrial cystoma of ovary rupture |  |  |  |
| Phase | First(n=15) Second (n=25) Third (n=165) | | | | | | | | | | | | |
| Case | 59 | 19 | 10 | 19 | 14 | 10 | 4 |  | 50 | 20 |  | F | p-value |
| Pre-operative | | | | | | | | | | | | | |
| Age | 32.42±6.17 | 44.21±4.77 | 49.40±5.36 | 31.53±7.1 | 49.07±3.63 | 49.40±5.36 | 30.25±1.50 | 40.86±9.77 | 34.34±6.58 | 31.10±4.13 | 33.41±6.14 | 25.58 | <0.0001 |
| BMI | 20.74±1.47 | 23.29±1.62 | 23.93±2.36 | 20.92±1.51 | 22.96±2.44 | 23.93±2.35 | 19.73±0.90 | 22.30±2.19 | 22.57±2.20 | 22.19±2.45 | 22.46±2.62 | 13.72 | <0.0001 |
| HB1 | 116.40±13.50 | 123.20±13.92 | 121.40±13.36 | 118.10±13.98 | 118.90±13.66 | 121.40±13.36 | 116.00±14.45 | 119.60±13.56 | 118.30±14.39 | 116.40±13.81 | 117.80±14.15 | 0.96 | 0.3895 |
| Intra-operative | | | | | | | | | | | | | |
| Operative time | 50.81±6.49 | 46.53±4.29 | 50.70±4.72 | 52.32±5.90 | 51.86±5.19 | 50.70±4.72 | 53.25±6.40 | 49.92±5.88 | 55.16±9.28 | 64.30±8.45 | 57.77±9.91 | 22.28 | <0.0001 |
| Fluid Infusion | 1.04±0.16 | 1.06±0.20 | 1.11±0.13 | 1.06±0.18 | 1.03±0.21 | 1.11±0.13 | 1.15±0.13 | 1.06±0.18 | 1.08±0.17 | 1.27±0.16 | 1.13±0.18 | 5.54 | 0.0046 |
| Estimated blood loss | 76.95±17.54 | 50.00±13.33 | 60.00±9.43 | 54.47±16.06 | 56.43±12.16 | 60.00±9.43 | 77.50±17.08 | 57.08±14.59 | 73.20±16.96 | 70.50±17.61 | 72.43±17.06 | 28.67 | <0.0001 |
| Incision Length at closure | 2.79±0.17 | 2.82±0.17 | 2.71±0.14 | 2.67±0.16 | 2.70±0.12 | 2.71±0.15 | 2.73±0.22 | 2.73±0.16 | 2.77±0.18 | 2.74±0.18 | 2.76±0.18 | 2.51 | 0.0835 |
| Post-operative | | | | | | | | | | | | | |
| HB 2 | 108.80±13.70 | 114.40±13.71 | 113.10±13.19 | 110.50±14.45 | 111.60±13.68 | 113.10±13.19 | 110.30±13.30 | 111.80±13.61 | 107.00±14.53 | 104.20±13.62 | 107.30±14.47 | 2.04 | 0.1321 |
| HB Drop | 7.59±2.17 | 8.79±1.27 | 8.30±1.70 | 7.63±1.67 | 7.28±1.73 | 24.30±3.43 | 5.75±1.50 | 7.79±1.75 | 11.34±2.29 | 12.20±2.24 | 11.59±2.29 | 81.51 | <0.0001 |
| Out of bed activity time | 24.61±2.94 | 24.47±2.63 | 24.30±3.43 | 25.89±2.47 | 24.71±3.41 | 24.30±3.43 | 22.75±1.50 | 24.74±2.93 | 24.62±3.02 | 26.85±2.78 | 25.26±3.11 | 0.88 | 0.4166 |
| Anal exhaust time | 25.00±3.50 | 23.84±3.11 | 25.30±3.4 | 25.89±2.40 | 24.43±2.93 | 25.30±3.40 | 25.00±4.16 | 24.87±3.02 | 24.54±3.17 | 25.00±3.28 | 24.67±3.18 | 0.17 | 0.8421 |
| Stay of hospitalization | 3.09±0.86 | 3.05±0.91 | 2.90±0.88 | 2.79±0.79 | 3.00±0.88 | 2.90±0.88 | 3.25±0.96 | 2.96±0.85 | 4.06±0.82 | 3.95±0.89 | 4.03±0.83 | 33.52 | <0.0001 |
| VAS | 2.95±0.75 | 3.11±0.81 | 3.10±0.88 | 3.21±0.92 | 2.64±0.74 | 3.10±0.88 | 3.50±0.58 | 3.05±0.84 | 2.84±0.82 | 3.10±0.79 | 2.84±0.82 | 1.06 | 0.3501 |

**Supplementary Table 5**. The patients’ demographic data and surgical outcome in adnexectomy (n=33) and myomectomy (n=74) performed by surgery A

|  | Adnexectomy | | | | | | Myomectomy | | | | |
| --- | --- | --- | --- | --- | --- | --- | --- | --- | --- | --- | --- |
| ICD-11 | 2F32.1 | 2F32.3 | GA10.2 | GA18.5 |  |  | 2E86.0 | | |  |  |
|  | Ovarian fibroma | Ovarian serous cystadenoma | Endometrial cystoma of ovary | Cystic of ovary torsion |  |  | Uterine leiomyoma | | |  |  |
| Phase | First(n=5) Second (n=5) Third (n=23) | | | |  |  | First | Second | Third |  |  |
| Case | 12 | 9 | 5 | 7 | F | p-value | 8 | 10 | 56 | F | p-value |
| Pre-operative | | | | | | |  | | | | |
| Age | 65.33±5.85 | 67.33±5.57 | 61.00±3.24 | 33.00±4.44 | 72.01 | <0.0001 | 37.13±6.49 | 37.40±6.77 | 41.75±6.68 | 3.06 | 0.0531 |
| BMI | 21.50±2.00 | 20.47±1.78 | 20.16±1.61 | 22.24±1.88 | 1.81 | 0.1666 | 23.05±2.21 | 22.47±2.54 | 22.28±2.34 | 0.39 | 0.6811 |
| HB 1 | 112.80±10.38 | 125.60±12.49 | 116.2±11.90 | 117.00±14.36 | 1.97 | 0.1405 | 113.40±19.73 | 114.50±16.87 | 117.60±13.12 | 0.43 | 0.6540 |
| Intra-operative | | | | | | |  | | | | |
| Estimated diameter in tumor volume |  |  |  |  |  |  | 5.88±0.99 | 7.50±1.27 | 7.77±1.95 | 3.85 | 0.0259 |
| Operative time | 49.92±5.73 | 48.33±4.90 | 50.00±6.89 | 49.71±4.99 | 0.17 | 0.9147 | 131.50±7.52 | 137.00±9.56 | 110.10±12.00 | 32.16 | <0.0001 |
| Fluid Infusion | 1.03±0.14 | 1.07±0.14 | 1.08±0.08 | 0.97±0.14 | 0.89 | 0.4559 | 1.33±0.15 | 1.15±0.18 | 1.06±0.18 | 8.01 | 0.0007 |
| Estimated blood loss | 45.83±10.84 | 46.67±14.14 | 46.00±15.17 | 45.71±12.72 | 0.01 | 0.9987 | 115.00±22.04 | 116.00±24.13 | 110.40±22.32 | 0.37 | 0.6941 |
| Incision Length at closure | 2.68±0.20 | 2.71±0.19 | 2.76±0.19 | 2.74±0.19 | 0.31 | 0.8182 | 3.08±0.16 | 3.12±0.24 | 2.92±0.16 | 7.14 | 0.0015 |
| Post-operative | | | | | | |  | | | | |
| HB 2 | 100.50±9.88 | 119.20±11.98 | 108.20±12.50 | 108.60±15.03 | 6.00 | 0.0022 | 100.30±19.73 | 103.00±17.58 | 105.30±13.71 | 0.46 | 0.63 |
| HB Drop | 12.25±2.67 | 6.33±2.45 | 8.00±2.35 | 8.43±1.13 | 12.21 | <0.0001 | 13.13±1.64 | 11.50±1.90 | 12.21±2.94 | 0.79 | 0.4574 |
| Out of bed activity time | 24.75±2.09 | 25.22±1.86 | 26.40±2.51 | 25.00±2.83 | 0.64 | 0.5939 | 25.75±2.12 | 26.40±3.00 | 26.14±2.78 | 0.12 | 0.8830 |
| Anal exhaust time | 28.25±2.49 | 27.78±2.39 | 28.80±3.27 | 28.71±2.69 | 0.24 | 0.8688 | 26.63±3.07 | 28.30±3.34 | 27.88±3.12 | 0.70 | 0.4996 |
| Stay of hospitalization | 4.58±0.51 | 4.67±0.50 | 3.80±0.45 | 3.57±0.53 | 9.26 | 0.0002 | 5.00±0.76 | 5.00±0.94 | 5.11±0.82 | 0.11 | 0.8938 |
| VAS in 24 hours | 2.92±0.90 | 2.89±0.78 | 2.20±0.45 | 3.58±0.53 | 1.09 | 0.3705 | 4.25±1.17 | 4.30±1.25 | 4.75±1.08 | 1.23 | 0.2989 |

**Supplementary Table 6.** The patients’ demographic data and surgical outcome in salpingectomy (n=19) and ovarian cystectomy (n=20) performed by surgery B

| ICD-11 | JA01.Z | | | | GA18.1 | | | | |
| --- | --- | --- | --- | --- | --- | --- | --- | --- | --- |
|  | Tubal pregnancy | | | | Corpus luteum hemorrhage or rupture | | | | |
| Phase | First | Second |  |  | First | Second | | |  |
| Case | 5 | 14 | t | p-value | 5 | 15 | t | p-value | |
| Pre-operative |  |  |  |  |  |  |  |  | |
| Age | 32.80±8.47 | 33.00±5.08 | 0.06 | 0.9502 | 29.60±9.56 | 33.13±7.02 | 0.89 | 0.3832 | |
| BMI | 22.22±2.22 | 22.06±1.66 | 0.17 | 0.8649 | 21.64±1.76 | 21.23±1.73 | 0.45 | 0.6563 | |
| HB 1 | 97.60±7.70 | 104.00±13.38 | 1.00 | 0.3313 | 100.00±7.81 | 109.20±13.52 | 1.43 | 0.1706 | |
| Intra-operative |  |  |  |  |  |  |  |  | |
| Operative time | 76.40±10.31 | 65.07±10.33 | 2.11 | 0.0504 | 72.80±6.38 | 67.00±5.81 | 1.89 | 0.0748 | |
| Fluid infusion | 1.18±0.11 | 1.00±0.16 | 2.35 | 0.0312 | 1.12±0.13 | 1.02±0.15 | 1.35 | 0.1947 | |
| Estimated blood loss | 34.00±11.40 | 55.71±12.22 | 3.46 | 0.0030 | 48.00±10.95 | 42.67±15.34 | 0.71 | 0.4848 | |
| Incision length at closure | 3.30±0.19 | 2.83±0.11 | 6.95 | <0.0001 | 3.32±0.11 | 2.87±0.10 | 8.71 | <0.0001 | |
| Post-operative |  |  |  |  |  |  |  |  | |
| HB 2 | 89.20±7.69 | 95.29±12.45 | 1.02 | 0.3243 | 92.40±7.57 | 100.10±13.07 | 1.23 | 0.2343 | |
| HB Drop | 8.40±1.95 | 8.71±2.95 | 0.22 | 0.8286 | 8.60±2.97 | 9.13±2.10 | 0.45 | 0.6616 | |
| Out of bed activity time | 25.20±3.11 | 24.93±2.30 | 0.81 | 0.4299 | 24.40±2.51 | 25.53±2.77 | 0.81 | 0.4299 | |
| Anal exhaust time | 24.80±4.76 | 26.21±2.81 | 0.64 | 0.5322 | 24.80±4.32 | 25.93±3.15 | 0.64 | 0.5322 | |
| Stay of hospitalization | 2.80±0.45 | 2.28±0.47 | 2.13 | 0.0482 | 2.80±0.45 | 2.60±0.51 | 0.78 | 0.4436 | |
| VAS | 2.80±0.84 | 3.71±1.20 | 1.56 | 0.1384 | 3.80±1.30 | 3.80±0.86 | 0.00 | >0.99 | |

Abbreviation and Unit for Supplementary Table 3-6：HSIL: high grade squamous intraepithelial lesion, Age (years), BMI (kg/m2), Hemoglobin 1(HB) (g/L), Estimated diameter in tumor volume (cm), Uterine Volume (gestational week), OP: Operative time (min), Fluid infusion (L), Estimated blood loss (ml), Incision Length at closure (cm), Out of bed activity time (hour), Anal exhaust time (hour), Stay of hospitalization (Days), VAS, Visual simulation scoring method, VAS in 24 hours. Data was presented as mean ± standard deviation (x ± s) with 95% confidence intervals (95% CI). Statistical significance was set at P<0.05. Demographic variables were compared between groups using chi-squared or Fisher's exact test. Perioperative data was analyzed using Student's t-test or single-factor ANOVA for continuous variables, and Mann-Whitney test for non-parametric variables.
